# Metagenomic shotgun sequencing and metabolomic profiling identify specific human gut microbiota associated with diabetic retinopathy in patients with type 2 diabetes

**DOI:** 10.3389/fimmu.2022.943325

**Published:** 2022-08-17

**Authors:** Lihua Li, Kaibo Yang, Cong Li, Han Zhang, Honghua Yu, Kang Chen, Xiaohong Yang, Lei Liu

**Affiliations:** ^1^Department of Ophthalmology, Affiliated Hospital of Weifang Medical University, Weifang, China; ^2^Department of Ophthalmology, The First Hospital of China Medical University, Shenyang, China; ^3^Guangdong Eye Institute, Department of Ophthalmology, Guangdong Provincial People’s Hospital, Guangdong Academy of Medical Sciences, Guangzhou, China

**Keywords:** diabetic retinopathy, gut microbiome, metagenomic, metabolomics, diabetes

## Abstract

**Background:**

Diabetic retinopathy (DR) is a common microvascular complication of diabetes mellitus (DM) and is one of the leading causes of blindness among DM patients. However, the molecular mechanism involving DR remains unclear.

**Methods:**

A case–control study with age-, sex-, and duration-matched diabetic patients and controls was conducted, which included 15 type 2 DM (T2DM) patients with DR and 15 T2DM patients without DR. Shotgun sequencing and non-targeted metabolomic profiling analyses of fecal samples were performed, and comprehensive bioinformatics analyses were conducted.

**Results:**

Using metagenomic analyses, we identified 293,460 unique genes in the non-DR group, while that in the DR group was 283,235, and the number of overlapping genes was 1,237,914. Regarding phylum levels, *Actinobacteria* decreased but *Bacteroidetes* increased in the DR group when compared with those in the control group. Regarding genus levels, *Bifidobacterium* and *Lactobacillus* decreased. Cellular processes, environmental information processes, and metabolism-related pathways were found at higher levels in the gut microbiome of DR patients. Using metabolomic analyses, we found 116 differentially expressed metabolites with a positive ion model and 168 differentially expressed metabolites with a negative ion model between the two groups. Kyoto Encyclopedia of Genes and Genomes annotation revealed six pathways with different levels between DR and diabetic controls, namely, cellular processes, environmental information processing, genetic information processing, human diseases, organismal systems and metabolism. Moreover, lysine biosynthesis and lysine degradation were enriched using a positive model, but histidine metabolism and β-alanine metabolism were enriched using a negative model.

**Conclusions:**

Together, the metagenomic profiles of DR patients indicated different gut microbiota compositions and characteristic fecal metabolic phenotypes in DR patients. Our findings of microbial pathways therefore provided potential etiological and therapeutic targets for DR patients.

## Introduction

Gut microbiota studies have recently become an intensive field of research. Under normal physiological conditions, the microbiome is a homeostatic ecosystem with several essential functions. Destruction of this ecosystem is called dysbiosis and is associated with multiple diseases. The composition and essential functional disorders of the gut microbiota are related to the pathophysiology of most chronic diseases, including obesity (1), cardiovascular system-related diseases (2), chronic kidney diseases (3), neurological diseases (4), and mental disorders (5). Recent studies have reported that the gut microbiota have been linked to type 2 diabetes mellitus (T2DM) (6, 7), and results have also suggested a basic mechanism of action.

Important insights have recently been reported regarding the possibility that gut microbiota associate with degenerative retinal diseases, including diabetic retinopathy (DR), uveitis, and age-related macular degeneration, supporting a “microbiota-gut-retina axis” (8). This implies that there is a connection between the gut microbiome and retinal diseases, with the composition and disturbance of the microbiome potentially regulating metabolism and acting as inflammatory or pathological factors in the retina.

DR involves microvascular complications of DM, which has become the leading cause of blindness and vision impairment in working-age adults (9). However, the potential mechanisms regarding DR pathogenesis remain unknown. The gut microbiota in DR etiology is also supported by alterations in species compositions and dysfunctions of the microbiome. Furthermore, metabolic factors related to chronic low-grade inflammation or oxidative stress that link an altered gut microbiota composition and T2DM may also affect the development of DR (10). For example, Beli et al. (11) reported that restructuring of the gut microbiome by long-term intermittent fasting prevented the development of DR and prolonged the life span by activation of tauroursodeoxycholate (TUDCA) receptor (TGR5) in a diabetic db/db mouse model. The recent focus on gut microbiome dysbiosis in DR patients has led to new pathophysiological and therapeutic directions (12–16). Most of these microbiome studies regarding DR used 16S rRNA sequencing, which limited their associations in the microbiome to the genus level. This knowledge gap can be addressed using whole-genome shotgun sequencing, which provides more in-depth taxonomic characterization and functional insights into human microbiomes (17). Metagenomics is a more accurate way to study the composition and interaction of cultured microorganisms in the sample; it can also identify metabolic pathways involving gene functions at the molecular level. However, no reports have used integrative metagenomics-metabolomics to identify possible relationships between various microbial compositions and functions in DR individuals. Herein, we used a 1:1 matched case–control study to identify undesirable biological genes/pathways and species-level clades of the human gut microbiome associated with DR by metagenomic shotgun sequencing and metabolomic profiling. The results may assist in the development of novel targeted therapies for the treatment of DR patients.

## Methods

### Subject recruitment and data collection

**Figure 1:**
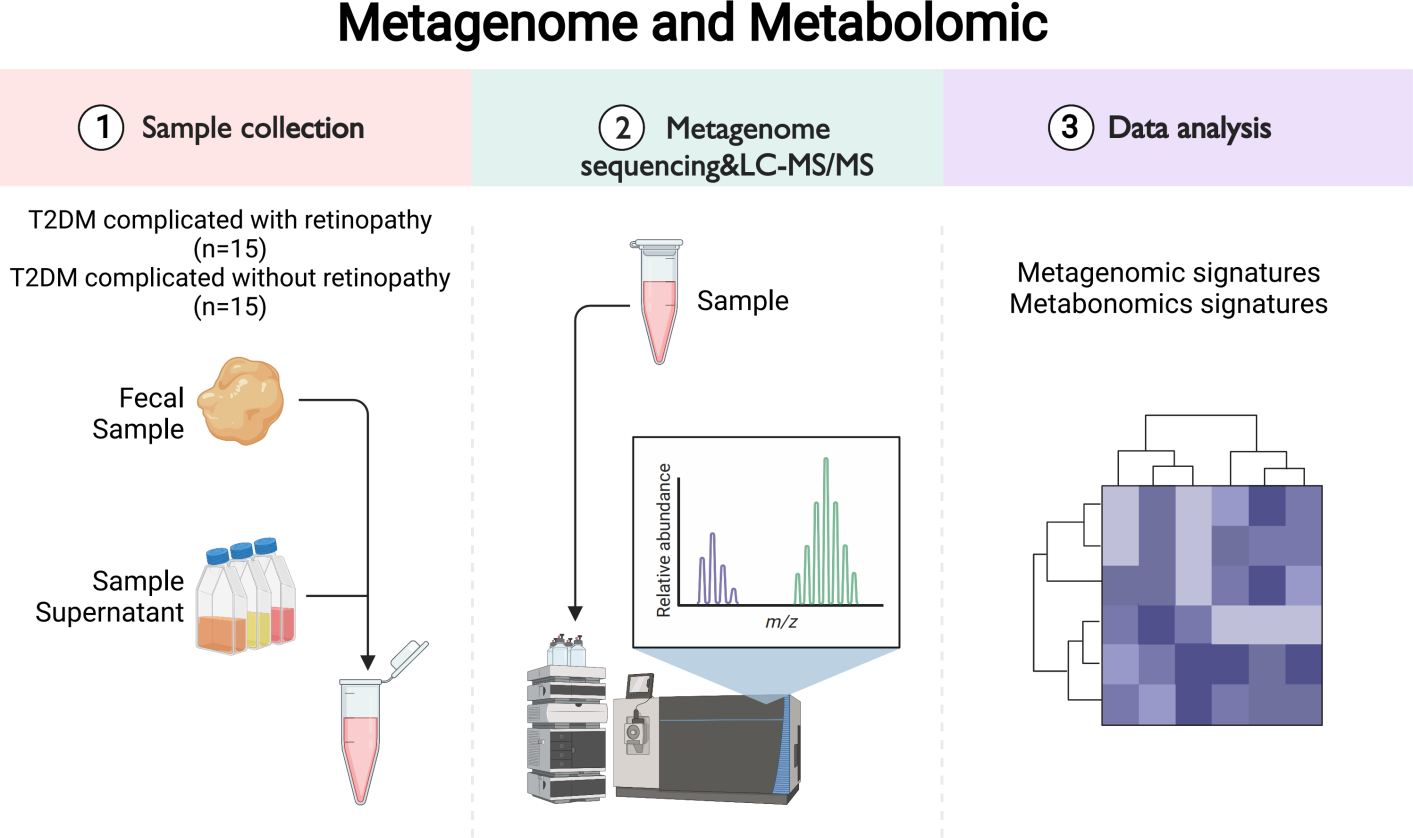
The research flowchart.

The research flowchart is shown in **Figure 1**. The institutional review board and the ethics committee at The First Affiliated Hospital of China Medical University approved this study (No. 201913). Written informed consent was obtained from all participants. In this 1:1 matched case–control study, 15 patients with T2DM complicated with retinopathy, aged >18 years, were recruited in the Endocrinology Department of the First Affiliated Hospital of China Medical University. Slit lamp videography, ophthalmoscopy, and fundus photography were used to determine the extent of fundus lesions. The diagnostic criteria of DR used the International Clinical Diabetic Retinopathy Disease Severity Scale (2002) (18). In addition, 15 patients with T2DM without any signs and symptoms of retinopathy were age-, sex-, and diabetes duration-matched and recruited from the same institution as the controls. Subjects who had a medical history of malignant tumors, severe abnormal liver functions, gastrointestinal diseases and gastrointestinal surgery, any infectious disease (e.g., HIV and syphilis), antibiotic/probiotic/prebiotic use, or known gastrointestinal symptoms such as diarrhea, constipation, abdominal pain, and blood-in-stool within a month were excluded from the study. Demographics, lifestyle (smoking status), and medical history information were collected using questionnaires on the day of stool sample collection. Diabetes duration was defined as the difference between age at study and age at onset. Current biochemical indicators were extracted from the medical records on the same day. Three peanut-sized stool samples were collected at the institution using DNA/RNA-free sterile swabs (BD BBL CultureSwab Sterile, Media-free Swabs kit; Fisher Scientific, Hampton, NH, USA) and stored immediately at 4°C for 12 h, transferred to -20°C for another 24 h, and then stored at -80°C until the DNA was extracted.

### Sample processing and shotgun metagenomic sequencing

Stool samples were thawed on ice and aliquoted, and genomic DNA was extracted using a Magnetic Soil and Stool DNA Kit (Tiangen, Beijing, China) according to the kit instructions. Quality check of DNA samples was performed using agarose gel electrophoresis to determine the extent of DNA degradation and potential contamination. DNA concentration was measured using a Qubit^®^ DS DNA Assay Kit (Thermo Fisher Scientific, Waltham, MA, USA) in a Qubit^®^ 2.0 Fluorometer (Life Technologies, Carlsbad, CA, USA). The absorbances were 1.8−2.0, with DNA contents >1 µg used to construct the library. A total of 30 stool samples were randomly assigned to extraction batches. To account for uncertain bacterial contamination during extraction, the PCR and sequencing kits included negative controls with each tissue DNA extraction batch (19).

Sequencing libraries were generated using a NEBNext^®^ Ultra™ DNA Library Prep Kit for Illumina (New England Biolabs, Ipswich, MA, USA) following the manufacturer’s recommendations. Index codes were added to attribute sequences to each sample. The qualified DNA samples were randomly disrupted using a Covaris M220 ultrasound apparatus (Covaris, Woburn, MA, USA), and the entire library was prepared after the fragments with a growth degree of about 350 bp were randomly disrupted. The library was then diluted to 2 ng/µl using a Qubit^®^ 2.0, and the insert size of the library was detected using an Agilent 2100 Bioanalyzer (Agilent, Santa Clara, CA, USA). After the correct insert size was obtained, the effective concentration of the library was quantified by PCR (the effective concentration of the library was set at >3 nM). PCR products were purified (AMPure XP system; Beckman Coulter, Brea, CA, USA), and libraries were analyzed for their size distribution using an Agilent 2100 Bioanalyzer and quantified using real-time PCR. After passing quality inspection, clustering of the index-coded samples was performed on a cBot cluster generation system according to Illumina PE150 instructions (Illumina, San Diego, CA, USA). After cluster generation, the library was ready to be sequenced on a NovaSeq 6000 platform (Illumina) using paired-end reads. All samples were sequenced on an Illumina platform in PE150 mode at Novogene Bioinformatics Technology (Beijing, China).

### Metagenomic data processing and analysis

Preprocessing the raw data obtained from the Illumina HiSeq sequencing platform using Readfq V8 was conducted to acquire clean data for subsequent analyses. Clean data were blasted using the host database, which defaulted using Bowtie2.2.4 (20) software to filter the reads that were of host origin. Metagenome assembly was conducted from the clean data of each sample after quality control, and the unused reads of each sample were combined for mixed assembly. For Scaftigs generated from single samples and mixed assemblies, fragments less than 500 bp were filtered and subjected to statistical analysis and subsequent gene prediction.

The clean data of each sample were mapped to an initial gene catalog using Bowtie2.2.4. The abundance information of each gene was calculated in each sample based on the number of mapped reads and the length of genes. The basic information statistics, core-pan gene analysis, correlation analysis of samples, and Venn diagram involving number of genes were all based on the abundance of each gene in each sample in the gene catalog.

DIAMOND V0.9.9 software was used to blast the unigenes to the sequences of bacteria, fungi, archaea, and viruses, which were extracted from the non-redundant protein sequence database of the National Center for Biotechnology Information (NCBI). A Lossless Compression Algorithm was used in the system classification of MEGAN software to ensure species annotation information of the sequences. Krona analysis, the relative abundance, the bioinformatics analyses of abundance cluster heat map, principal coordinate analysis (PCoA) and non-econometric multidimensional (NMD) scaling analysis (21), and decrease-dimension analyses were based on the abundance table of each taxonomic hierarchy. The difference between groups was tested by analysis of similarities (ANOSIM). Metastat and the Linear discriminant analysis (LDA) Effect Size (LEfSe) (22) analysis were used to identify different species between two groups.

DIAMOND V0.9.9 software (23) was used to blast Unigenes to identity functional databases with the parameter settings of the blasts. The functional databases included the Kyoto Encyclopedia of Genes and Genomes (KEGG) Orthology catalog database, the eggNOG database, and the CAZy database. Based on the abundance table of each taxonomy hierarchy, the exhibition of the general relative abundance situation, exhibition of the abundance cluster heat map, and the decrease-dimension analysis of PCoA and NMDS were conducted. In addition, the ANOSIM (24) test of the differences between cases and control individuals based on functional abundance, the comparative analysis of metabolic pathways, and the MetaStat and LEfSe analyses of functional differences between two groups were conducted.

### Non-targeted metabolomic analysis of fecal samples

A total of 50 mg of stool sample was placed in an Eppendorf tube for metabolite extractions, followed by addition of 1,000 µl extract solution (acetonitrile:methanol:water = 2:2:1). After vortexing, the samples were homogenized at 35 Hz and sonicated. The supernatants were then obtained after incubation and centrifugation. The resulting supernatant was prepared for quality control and analysis. Liquid chromatography-tandem mass spectrometry was conducted using an ultra-high-performance liquid chromatography (UHPLC) system (Vanquish; Thermo Fisher Scientific) with an ultra performance liquid chromatography (UPLC) BEH Amide column (2.1 mm × 50 mm; 1.7 µm; Waters, Milford, MA, USA) coupled to a QExactive HFX mass spectrometer (Orbitrap Ms; Thermo Fisher Scientific). The QE HFX mass spectrometer was used to acquire MS/MS spectra using an information-dependent acquisition mode under the control of the acquisition software (Xcalibur; Thermo Fisher). In this mode, the acquisition software continuously evaluated the full scan mass spectra. The raw data were converted to the mzXML format using ProteoWizard and processed with an in-house program, which was developed using R software (R Project for Statistical Computing, Vienna, Austria). An in-house MS2 database (BiotreeDB) was then used for metabolite annotation. For three fecal samples, all technical experiments were replicated three times to acquire robust results. During raw data preprocessing, based on the relative standard deviation filtering deviation values, only the peak area data with no more than 50% null values or no more than 50% null values were reserved, and the missing values in the raw data were simulated with a minimum half of the value. Total ion current was then used with each sample for normalization.

### Correlation analysis of gut microbial species and metabolites

We selected gut microbial species discriminately enriched in the DR or control groups by LEfSe analysis, with LDA >3 and P < 0.05. Significantly abundant metabolites were defined as log_2_fold change (FC) >1 or <-1, P < 0.05, q < 0.05, with 31 metabolites included. Spearman’s correlation of differentially enriched species and metabolites was calculated using the scipy-stats package. Heat maps were hierarchically clustered to represent the species-metabolite-associated patterns based on the correlation distance.

### Statistical analysis

Differences in clinical indices among two groups were determined using Student’s *t*-test or the Kruskal–Wallis test. Differentially elevated or depleted gut microbes and fecal metabolites were evaluated using the Wilcoxon rank sum test. The connection of microbes to host metabolites was assessed using Spearman’s rank correlation, and the importance was corrected using the Benjamini–Hochberg procedure. Differentially enriched KEGG modules/pathways were identified according to their reporter scores, which were calculated from the Z-scores of individual KEGG orthologous (KO) groups (25). A module with reporter score of Z >1.5 (>90% confidence according to a normal distribution) was considered as a significant dysbiosis module (3). All data analyses were conducted using the R Statistical Computing framework v3.4 (The R Project for Statistical Computing). All statistical tests were two-tailed, and a P < 0.05 was considered statistically significant.

## Results

The study recruited 15 DR patients and 15 diabetic controls without DR (NDR). Participant demographics, lifestyles, and clinical information are shown in **Table 1**. There was no significant difference regarding age, sex, and medical indicators and biochemical test results between the NDR and DR participants. To examine the gut microbiome, 30 fecal samples were processed, and the DNA was extracted and sequenced using whole-genome shotgun sequencing.

**Table 1:** Demographic and clinical characteristics of diabetic patients with and without DR.

	Total (n = 30)	Diabetic controls (n = 15)	DR (n = 15)	P value
Age, years	57 (51–62)	57 (51–62)	55 (51–63)	0.86
Sex (men), %	15 (50.0)	7 (46.7)	8 (53.3)	0.72
BMI, kg/m2	26.6 (24.8–29.0)	27.6 (25.5–30.3)	26.0 (23.5–28.0)	0.20
Diabetes duration, years	12 (9–15)	10 (9–14)	13 (8–17)	0.39
Smoking (yes), %	3 (10.0)	2 (66.7)	1 (33.3)	0.55
HbA1c, %	8.0 (7.2–10.0)	7.8 (6.9–9.5)	8.7 (7.5–10.4)	0.19
SBP, mm Hg	149 (134–160)	140 (131–156)	157 (138–165)	0.52
DBP, mm Hg	85 (77–90)	84 (78–90)	85 (74–89)	0.56
TG, mmol/L	1.7 (1.5–2.9)	1.7 (1.6–3.0)	1.6 (1.4–2.8)	0.65
TC, mmol/L	4.8 (4.1–5.3)	4.8 (3.7–5.6)	4.7 (4.5–5.1)	0.92
HDL, mmol/L	1.0 (0.9–1.2)	1.0 (0.9–1.1)	1.1 (1.0–1.3)	0.24
LDL, mmol/L	2.8 (2.3–3.5)	3.2 (2.1–3.8)	2.8 (2.5–3.3)	0.67
SCr, μmol/L	54 (40–63)	53 (40–62)	57 (40–63)	0.70
BUN, mmol/L	6.1 (4.9–7.1)	5.8 (5.1–6.8)	6.5 (4.6–7.2)	0.47
ApoA-1, g/L	1.2 (1.1–1.4)	1.2 (1.1–1.3)	1.3 (1.2–1.4)	0.19
ApoB, g/L	1.0 (0.8–1.2)	1.1 (0.8–1.2)	1.0 (0.9–1.1)	0.51
LP(a), nmol/L	22.7 (10.3–66.9)	37.4 (6.2–68.4)	21.8 (19.2–53.3)	0.34
Cys-c, mg/L	0.9 (0.8–1.0)	0.9 (0.8–1.0)	1.0 (0.9–1.2)	0.12
eGFR, ml/min/1.73 m2	108.4 (101.4–113.7)	109.9 (102.8–113.0)	106.4 (100.5–114.4)	0.86
CK, U/L	76.5 (57.5–95.5)	72.0 (54.0–94.0)	92.0 (67.0–100.0)	0.39
LDH, U/L	176.5 (150.0–191.0)	166.0 (135.0–188.0)	178.0 (172.0–194.0)	0.13
UA, μmol/L	322.0 (275.0–404.0)	323.0 (251.0–404.0)	311.0 (293.0–399.5)	0.76
FT4, pmol/L	13.3 (12.4–13.8)	13.3 (12.4–13.7)	12.9 (12.4–14.1)	0.54
FT3, pmol/L	4.2 (4.0–4.6)	4.2 (4.0–4.6)	4.2 (4.0–4.7)	0.47
TSH,µIU/ml	1.7 (1.2–2.7)	2.3 (1.3–3.3)	1.7 (0.9–2.0)	0.07
TPOAb, IU/ml	0.4 (0.1–1.4)	0.4 (0–116.1)	0.5 (0.2–0.8)	0.40
TGAb, IU/ml	1.6 (0.8–3.3)	1.7 (0.9–6.1)	1.2 (0.8–2.6)	0.52

Categorical variables are presented as counts (percentages), continuous variables are presented as median [interquartile range (IQR)].

ApoB, apolipoprotein B; APOA-1, apolipoprotein A1; BMI, body mass index; BUN, blood urea nitrogen; Cys-c, cystatin C; CK, creatine kinase; DR, diabetic retinopathy; DBP, diastolic blood pressure; eGFR, estimated glomerular filtration rate; FT4, free thyroxine 4; FT3, free thyroxine 3; HbA1c, glycosylated hemoglobin; LDH, lactate dehydrogenase; LP(a), lipoproteins; TG, triglyceride; TC, total cholesterol; TSH, thyroid-stimulating hormone; TPOAb, thyroid peroxidase antibody; TGAb, tnti-thyroglobulin antibodies; SBP, systolic blood pressure; SCr, serum creatinine; UA, uric acid.

### Metagenomic signature data of diabetic retinopathy (DR) and diabetic retinopathy (NDR) participants

**Figure 2:**
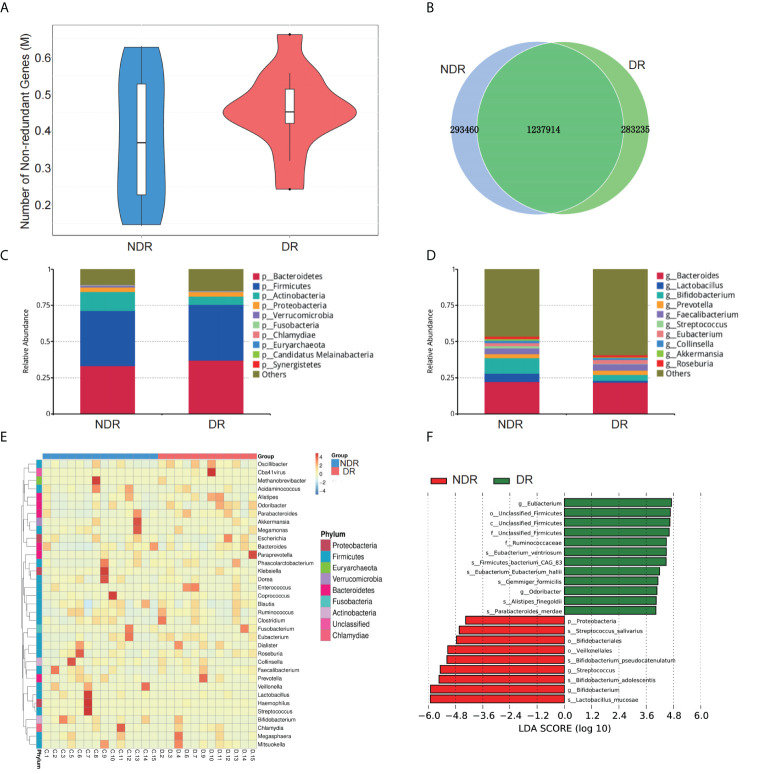
Characterization of the gut microbiome from metagenomic data. **(A)** The number of nonredundant genes in the DR and NDR groups. **(B)** The identification of genes in the DR and NDR groups. **(C)** Top 10 microbiome phyla between the case and control groups. **(D)** Top 10 microbiome from the genera of case and control groups. **(E)** Heat map showing the levels of microbiome from each participant. **(F)** The influence of differentially expressed microbiome using LDA. DR, diabetic retinopathy; NDR, diabetes without diabetic retinopathy; LDA, linear discriminant analysis.

The number of genes in the gene catalog was 1,957,836. The total length of genes in the gene catalog was 1,470.48 Mbp. The mean length of genes in the gene catalog was 751.25 bp, and the guanine to cytosine (GC) percentage was 47.45%. The basic characteristics of genes in all samples are shown in **Supplementary Figure S1**. The gene catalog length distribution statistics are shown in **Supplementary Figure S1A**, and the core-pan gene dilution curves are shown in **Supplementary Figures S1B, C**. The numbers of nonredundant genes in the DR and control participants are shown in **Figure 2A**. The number of unique genes in the NDR group was 293,460, while that in the DR group was 283,235, and the number of overlapping genes was 1,237,914 (**Figure 2B**). In addition, we analyzed the top 10 predominant bacterial genera (at high abundances >5%) regarding phylum and genus (**Figures 2C, D**) between DR and NDR participants and found that at the phylum level, *Actinobacteria* decreased but *Bacteroidetes* increased. At the genus level, *Bifidobacterium* and *Lactobacillus* decreased in the DR group when compared with those in the control group (Wilcoxon test, P < 0.005). Cluster analyses using a heat map indicating the level of microbiome in each participant are shown in **Figure 2E**. Furthermore, LDA indicated that *Eubacterium* (LDA = 4.71) had the highest score in the DR group, and *Lactobacillus mucosae* (LDA = 5.91) had the highest score in the control group (all LDA >3, P < 0.005) (**Figure 2F**).

**Figure 3:**
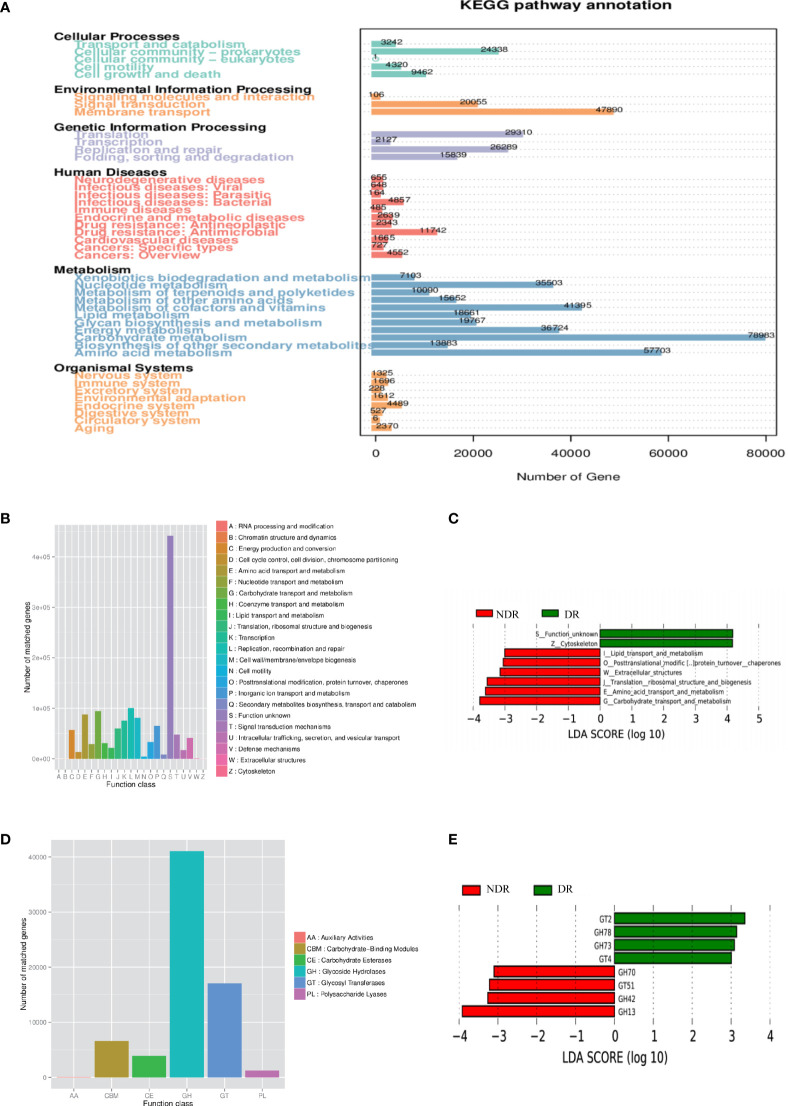
Characterization of the gut microbiome from function annotation. **(A)** The unigene number statistics of the Kyoto Encyclopedia of Genes and Genomes. **(B)** The unigene number statistics of eggNOG. **(C)** The LEfSe analysis of the functional differences between the groups of eggNOG. **(D)** The unigenes number statistics of CAZy. **(E)** The LEfSe analysis of functional differences between the groups of CAZy.

Furthermore, we also conducted bioinformatics analyses regarding the abundance tables at various taxonomic levels to identify their potential roles in the etiology of DR. ANOSIM was performed based on the functional abundances of the KO, Level 1 of eggNOG, and Level 2 hierarchy of CAZy, but none of the differences between groups were statistically significant (**Supplementary Figure S2**). **Figure 3A** shows the number of unigenes of the KEGG. Regarding the Metastat of functional differences between groups, we found that cellular processes, genetic information processes, immune diseases, and metabolism-related pathways were enriched, suggesting that these pathways had important roles in the process of DR (**Table 2**). Among them, metabolism-related pathways included carbohydrate metabolism, amino acid metabolism, metabolism of other amino acids, biosynthesis of other secondary metabolites, lipid metabolism, and energy metabolism. Regarding eggNOG analyses, **Figure 3B** shows the number of unigenes and the LEfSe analyses of functional differences between the groups (**Figure 3C**), which showed that several metabolism-related pathways were enriched. Among them, the metabolism-related pathways included lipid transport and metabolism, amino acid transport and metabolism, and carbohydrate transport and metabolism. Regarding CAZy analyses, **Figure 3D** shows the number of unigenes and the LEfSe analyses of the functional differences between the groups (**Figure 3E**), which showed that two classes of carbohydrate enzymes were enriched, including glycoside hydrolases and glycosyl transferases.

**Table 2:** Metastat of the functional differences between the groups.

KEGG level 2	Mean (NDR)	Standard error (NDR)	Mean (DR)	Standard error (DR)
Metabolism; Carbohydrate metabolism	0.045055	0.001350	0.039049	0.002067
Cellular Processes; Cell growth and death	0.005582	0.000174	0.004887	0.000237
Human Diseases; Immune diseases	0.000351	0.000330	0.000273	0.001231
Metabolism; Metabolism of other amino acids	0.009551	0.000403	0.008123	0.000471
Genetic Information Processing; Folding	0.009485	0.000301	0.008408	0.000397
Human Diseases; Infectious diseases: Bacterial	0.002702	0.000140	0.002324	0.00012
Genetic Information Processing; Transcription	0.001274	0.000131	0.000970	0.007061
Metabolism; Amino acid metabolism	0.034519	0.001297	0.030546	0.001477
Metabolism; Biosynthesis of other secondary metabolites	0.008683	0.000271	0.007653	0.000399
Metabolism; Lipid metabolism	0.010915	0.000429	0.009629	0.000478
Organismal Systems; Aging	0.001551	0.007120	0.001341	0.000753
Metabolism; Energy metabolism	0.021537	0.000578	0.019669	0.000859

KEGG, Kyoto Encyclopedia of Genes and Genomes; DR, diabetic retinopathy; NDR, diabetes without diabetic retinopathy.

### Metabonomics signatures of DR and NDR participants

**Figure 4:**
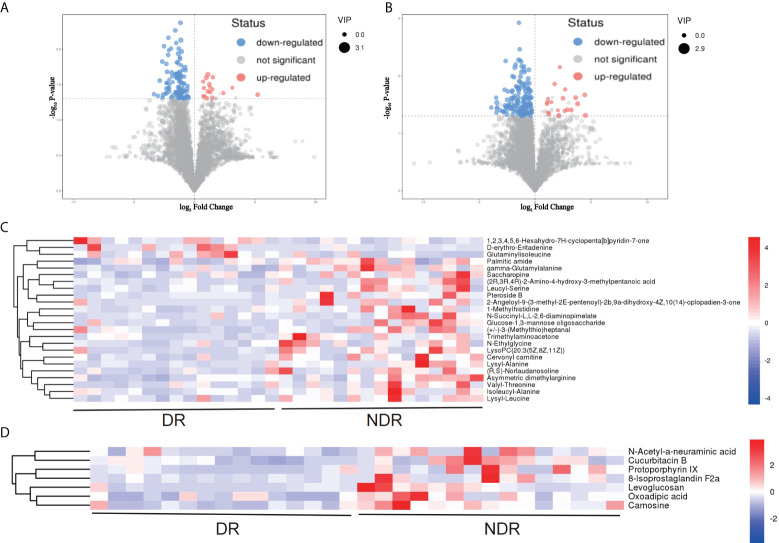
DE metabolites between DR and NDR. **(A)** Volcano plot indicating upregulated and downregulated metabolites using the positive ion model. **(B)** Volcano plot indicating upregulated and downregulated metabolites using the negative ion model. **(C)** Heat map of DE metabolites using the positive ion model. **(D)** Heat map of DE metabolites using the negative ion model. DE, differentially expressed.

**Figure 5:**
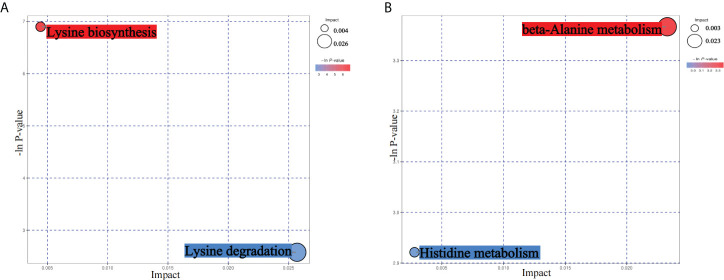
Bioinformatics analyses of differentially expressed (DE) metabolites. **(A)** Kyoto Encyclopedia of Genes and Genomes (KEGG) pathway analyses of DE metabolites using the positive ion model. **(B)** KEGG pathway analyses of DE metabolites using the negative ion model.

From the results of metagenomics of DR patients and controls, bioinformatics analyses suggested that microbial metabonomics pathways could play an important role in DR. We therefore conducted metabonomics analyses using fecal samples to identify the role of metabolism in DR. All samples had good qualities and system stabilities. The principal component analysis score plots (26) of the Quality control (QC) samples with positive and negative ions, respectively, are shown in **Supplementary Figure S3**. We identified differentially expressed (DE) metabolites using significance analysis with projection (VIP) values >1 and P < 0.05. As a result, we detected 116 DE metabolites using the positive ion model and 168 DE metabolites using the negative ion model (**Figures 4A, B**). The expression pattern of DE metabolites using positive and negative ion models is shown using a cluster heat map (**Figures 4C, D**). The radar plot of DE metabolites is shown in **Supplementary Figure S3**. Furthermore, bioinformatics analyses to detect the potential roles of DE metabolites revealed that lysine biosynthesis and lysine degradation were enriched using the positive model (**Figure 5A**), while histidine and β-alanine metabolisms were enriched using the negative model (**Figure 5B**), suggesting that the lysine-related metabolism, histidine metabolism, and β-alanine metabolism could play important roles in the etiology of DR.

### Analysis of metagenomic and metabolomic associations of DR and NDR participants

**Figure 6:**
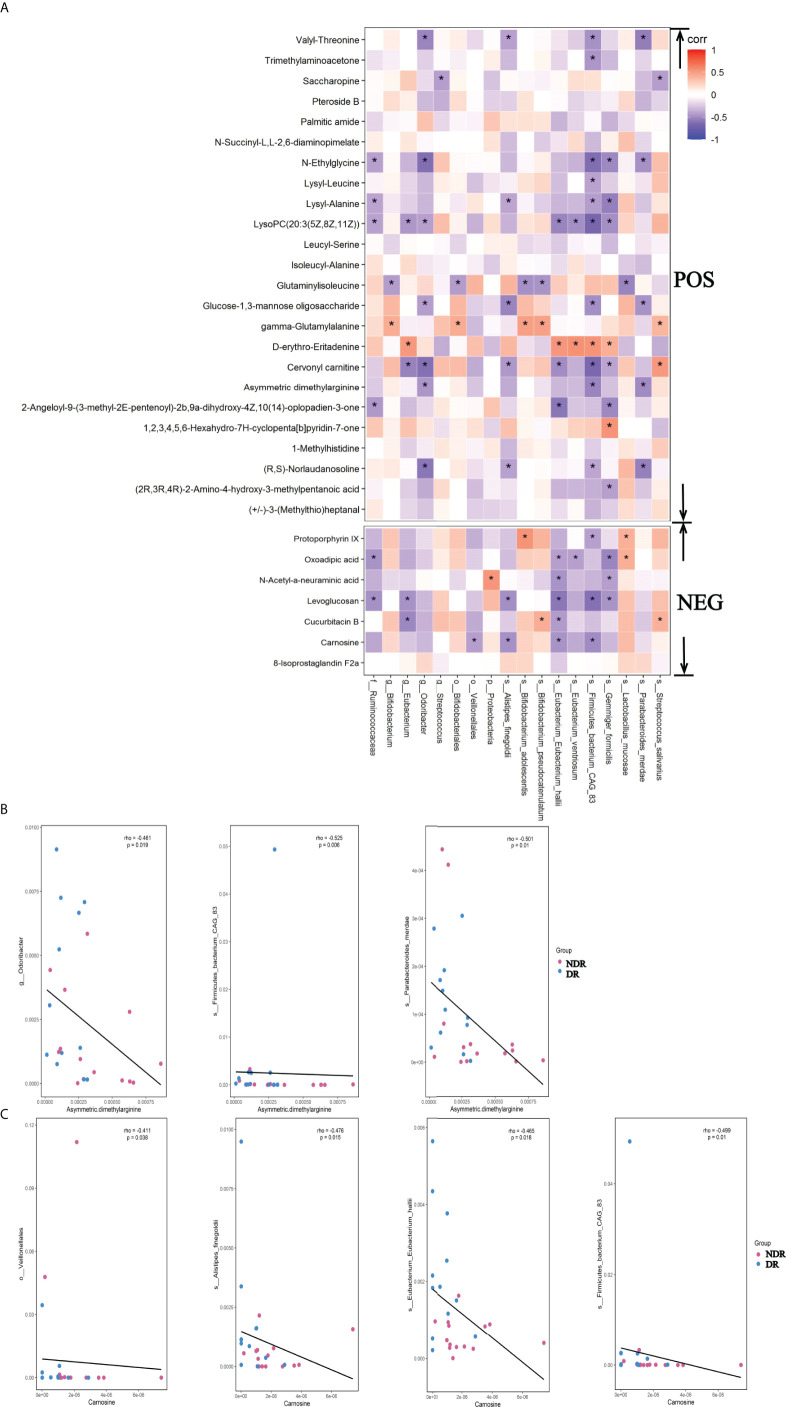
Metagenomic and metabolomic association between DR and NDR. **(A)** Heat maps were hierarchically clustered to represent the species metabolite-associated patterns based on the correlation distances. **(B)** The scatter plots of asymmetric dimethylarginine in the positive ion model with the associated microflora (P < 0.05). **(C)** The scatter plots of carnosine in the negative ion model with the associated microflora (P < 0.05). *P<0.05.

To identify the association of metagenomes and metabonomics of DR patients and NDR patients, we correlated the results from LEfSe analyses of divergent species between the metagenomes and the differential metabolites in the metabonomics. We found 17 metabolites associated with the divergent species using the positive ion model and six metabolites associated with the divergent species using the negative ion model (**Figure 6A**, P < 0.05), suggesting that divergent species between gut microbiota may ultimately affect the pathophysiology of DR patients by affecting intestinal metabolic processes. The scatter plots of asymmetric dimethylarginine in the positive ion model with the associated microflora Odoribacter, Firmicutes-bacterium-CAG83, and Parabacteroides-merdae (P < 0.05) are shown in **Figure 6B**. Simultaneously, the scatter plots of carnosine in the negative ion model with the associated microflora Veillonellales, Alistipes-fineg, Eubacterium-Eubacterium-hallii, and Firmicutes-bacterium-CAG83 (P < 0.05) are shown in **Figure 6C**.

## Discussion

DR is a common and specific microvascular complication of diabetes and remains the leading cause of preventable blindness in the working-age population (9). However, the exact molecular mechanisms of DR remain unclear. In our current study, we investigated the associations of the gut microbiome with DR among Chinese patients with T2DM using metagenomic shotgun sequencing combined with metabolomics profiling and a well-designed case–control study that controlled for potential confounders. This study provided metagenomic insight into the gut microbiome in DR patients and identified a cluster of DR subjects whose gut microbiome was different from that of the NDR group. It was of interest to find *Eubacterium* enriched in the gut microbiome of DR patients. Moreover, microbial pathway analysis indicated that there were three pathways in the gut microbiome with higher relative abundance levels in DR patients when compared with the NDR group.

Next-generation high-throughput sequencing and mass spectrometry have been increasingly used to identify disease mechanisms using metagenomic and metabonomics data (27, 28), suggesting that the gut microbiome and metabolites could play important roles in the pathological processes of different diseases. However, to the best of our knowledge, there has been no such study for DR, which could provide important clues regarding the molecular mechanisms of DR. We therefore conducted metagenomic and metabonomics studies using fecal samples from DR and NDR participants. Based on bioinformatics analyses, we found that metabolism-related pathways were enriched in metagenomic results, suggesting that the DE gut microbiome could influence metabolism processes in DR patients when compared with the NDR group. Regarding the metabonomics results, we found that lysine-related metabolism, histidine metabolism, and β-alanine metabolism were enriched, suggesting that these aforementioned metabolism-related pathways could play important roles in the pathogenesis of DR. Recent single-cell RNA sequencing for DR showed that Müller cell-mediated changes in β-alanine and histidine signaling were important for pathway and cell-specific alterations in the progression of DR, which also supported our conclusions (29).

Studies have suggested an association between human gut microbiota dysbiosis and DR. There is a significant increase in the percentage of *Bacteroidetes* in DR patients when compared with healthy controls. Higher DR *Bacteroidetes* abundance has also been reported in previous 16S rRNA sequencing studies (12). Unlike these studies, we found a decrease in the DR group at the genus level of *Bifidobacterium* and *Lactobacillus*. *Lactobacillus* is a common probiotic bacterium with good immunomodulatory and antioxidant properties (30), and its elevated levels have been positively correlated with long-term control of fasting blood glucose, glycosylated hemoglobin, and glycemic control (20). *Bifidobacterium* can inhibit harmful bacteria, improve gastrointestinal barrier function, inhibit the release of pro-inflammatory cytokines (31), increase the production of short-chain fatty acids, and affect the metabolic transformation of intestinal microbiota (32). Our results suggested that a reduction of *Lactobacillus* and *Bifidobacterium* may be involved in the mechanism of DR. Nevertheless, a significant increase in *Acidaminococcus*, *Escherichia*, and *Enterobacter* was observed in patients with DR (14). A recent study showed that a significant decrease in 18 genera was observed in DR patients, and 12 of 18 genera were also decreased in DM (13). Analysis of microbiota composition showed a significant decrease in the percentage of *Pasteurellaceae* in DR patients (12). At the genus level using high-throughput 16S rDNA analysis, *Faecalibacterium*, *Roseburia*, *Lachnospira*, and *Romboutsia* were enriched in DR patients (16). The 16S rRNA gene sequencing and untargeted metabolomics showed that reduced diversity and altered composition of gut microbiota and specific microbe–metabolite interplay were associated with proliferative DR (15). These previous studies in humans linking the gut microbiome to DR reached divergent conclusions and differed from our findings (12–16). However, it was difficult to directly compare these findings due to different study designs, sequencing techniques, statistical methods, and varied confounders. In the present study, we conducted a case–control study (age, sex, and diabetic duration were all matched), which included a comprehensive analysis of systematic variables as potential confounders. Moreover, we used both metagenomic shotgun sequencing and metabolomic profiling to ensure that outcomes were robust and further discovered the role of the gut microbiome on DR at global, taxonomic, and functional levels. Our findings complemented and may coalesce seemingly inconsistent results from various reports.

A key question is the role of microbiome dysbiosis in the etiology of DR. Gut biodiversity and its function in subjects with metabolic disorders have been particularly concerned with type 1 diabetes and T2DM complications (33). A wide spectrum of scientific research has shown how the gut microbiome influences the host through different pathways, including products of gut microbial metabolites (33). Microbial metabolites are key mediators of microbe–host crosstalk, significantly affecting the organism’s glucose metabolism. Intestinal microbiota produces metabolites such as short-chain fatty acids, amino acids, trimethylamine N-oxide, bile acids, and indole propionic acids, which participate in the regulation of host metabolism and gut integrity (34, 35). Using these metabolites, the gut microbiome moderates and generates its complex effects on diseases such as diabetes. As a complication of diabetes, a previous study reported that DR patients had higher bacterial conjunctival flora when compared with T2DM patients without DR (36), which suggested that the gut microbiome could be associated with DR. However, the exact role of the gut microbiome in DR remains largely unknown. Our study highlighted the key roles of the gut microbiome in DR, but further studies are still needed to identify the molecular mechanisms involved in the process of DR.

Although the above findings suggested the association of gut microbiota with DR, the altered gut microbiota and its pathways in host-modulating DR conditions remain unclear. In the present study, we identified pathways involved in the differential abundance of metabolites between DR patients and NDR patients. Importantly, lysine acetylation was one of the most widespread posttranslational modifications (PTMs) and induced modifications such as succinyl-lysine, malonyl-lysine, and acetyl-lysine (37, 38). Furthermore, the succinylome of the vitreous humor is significantly enriched in the regulation of defense responses during DR (39). Different types of protein modifications could change the activity of proteins and affect different pathways during disease progression. Using metabonomics analyses, we found that lysine biosynthesis and lysine degradation were enriched in the host during DR, suggesting that different types of PTMs might have changed during the pathophysiological processes of DR. However, little is known regarding the potential roles of PTMs in DR when compared with NDR, which could explain the molecular mechanisms of DR. Carnosine was a differential metabolite that we identified in the negative model. Recent studies have found that it improved DR through the Mitogen-activated protein kinases/extracellular regulated protein kinases (MAPK/ERK) pathway (40) and that carnosine and its derivatives were possible novel treatments for diabetic vascular complications (41). We found a significant correlation between carnosine and the gut microbial *Eubacterium hallii*. *E. hallii* is a major producer of short-chain fatty acids, providing an energy source for enterocytes and inducing anti-inflammatory effects in the intestinal tract to enhance the function of the intestinal barrier (42). Previous studies showed that *E. hallii* also contributed to the formation of intestinal propionate and cobalamin (43). Propionate was identified as a health-promoting intestinal metabolite (44), and cobalamin was beneficial for neuropathy in DR patients (45). These results suggested a potential beneficial effect of *E. hallii* in DR patients. Because the asymmetric dimethylarginine metabolite inhibited intercellular communication in retinal pericytes, it could aggravate the destruction of the blood–retinal barrier in DR and contribute to neovascularization in DR patients (46, 47). From our correlation analysis, the associated bacteria were Odoribacter, CAG83, and merdae, whose effects need future experimental verification (48). Further research on the exact mechanisms of the gut microbiota and metabolites in DR patients is also needed, which might mechanistically be involved in DR.

The main strength of this study was that it was the first to identify the mechanisms of human fecal microbiota during DR using both metagenomic and metabonomics analyses. In addition, enrollment of participants was based on strict inclusion/exclusion criteria, and the recruited subjects were strictly control matched, indicating that the stool samples in the current study were reliable and our findings were repeatable. Several limitations should also be addressed. First, we did not perform validations of molecular mechanisms *in vivo* or *in vitro*, which are now our future research objectives. Second, the sample size was relatively small due to the use of strict inclusion and exclusion criteria. Third, we did not collect information on dietary habits, which may have influenced the gut microbial composition. Finally, due to the inherent limitations of a case–control study, we could not reveal the causal relationships between identified differential gut microbial and DR; thus, further large-scale prospective studies are still needed. Based on the present and future studies, it is hoped that probiotic products can be developed for the prevention and treatment of DR patients.

## Conclusion

The use of metagenomics and metabonomics analyses has highlighted the relationships of gut microbiomes in DR patients. This study identified differential gut microbiota compositions and characteristic fecal metabolic phenotypes, as well as pathways with differentially abundant metabolites in DR patients, when compared with those in patients with T2DM. Our findings may provide new insights into the pathophysiology of DR and the basis for the development of novel treatments for DR patients.

## Data availability statement

The datasets presented in this study can be found in online repositories. The names of the repository/repositories and accession number(s) can be found below: https://www.ncbi.nlm.nih.gov/sra PRJNA855128.

## Ethics statement

This study was approved by Ethic Committee at The First Affiliated Hospital of China Medical University approved this study (No.[2019]13). The patients/participants provided their written informed consent to participate in this study.

## Author contributions

LL, XY, and KC contributed to the conception and design of the study, analyzed the data, and edited the manuscript. LHL, KY, CL, HZ, and HY performed experiments, and wrote and edited the manuscript. All authors contributed to the article and approved the submitted version

## Funding

This study was supported by Science and Technology Program of Guangzhou, China (202002020049) (XY); Project of Special Research on Cardiovascular Diseases (2020XXG007) (XY) and GDPH Supporting Fund for Talent Program (LL).

## Conflict of interest

The authors declare that the research was conducted in the absence of any commercial or financial relationships that could be construed as a potential conflict of interest.

## Publisher’s note

All claims expressed in this article are solely those of the authors and do not necessarily represent those of their affiliated organizations, or those of the publisher, the editors and the reviewers. Any product that may be evaluated in this article, or claim that may be made by its manufacturer, is not guaranteed or endorsed by the publisher.
