# Supplementary material for: Metagenomic shotgun sequencing and metabolomic profiling identify specific human gut microbiota associated with diabetic retinopathy in patients with type 2 diabetes
Source: Front Immunol. 2022 Aug 17;13:943325. doi: 10.3389/fimmu.2022.943325 (PMC9434375; doi:10.3389/fimmu.2022.943325)
Supplement: Supplementary file 1 [file DataSheet_1.docx]

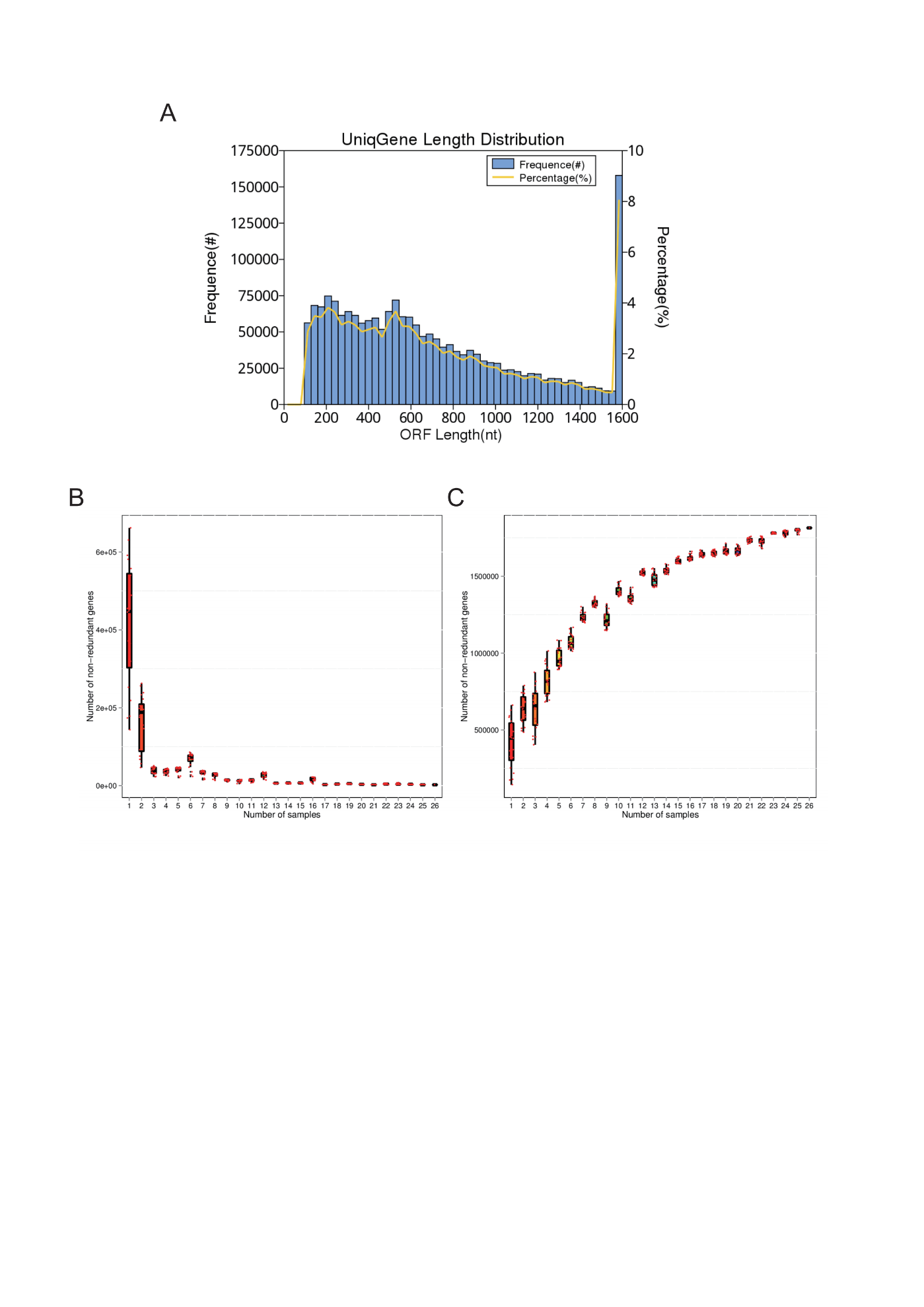


**Supplementary Figure 1.** A-C. Basic characteristics of metagenomic data.


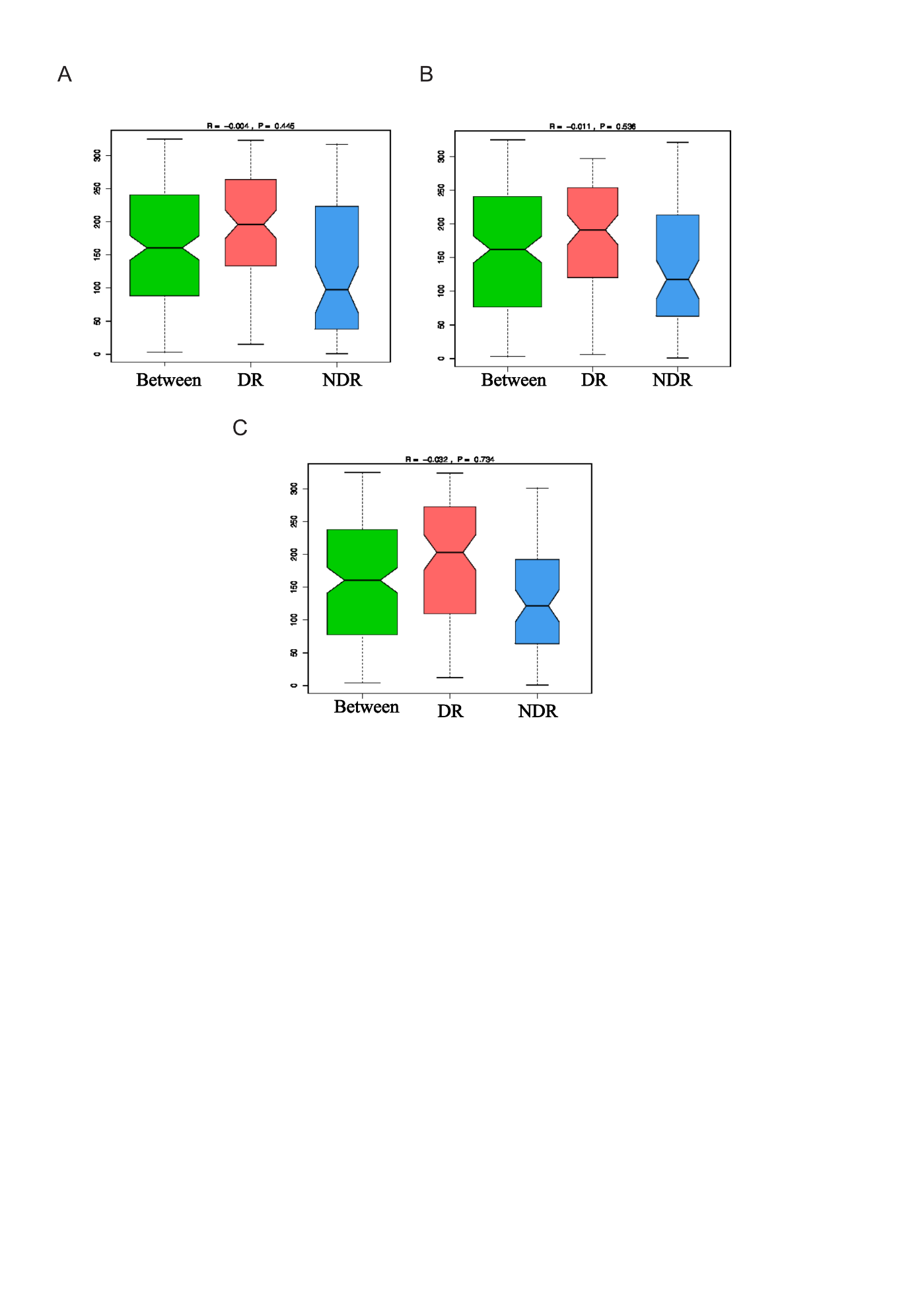


**Supplementary Figure 2.** The Anosim analysis the functional abundances. (A) Ko of KEGG. (B) The level1 of eggNOG. (C) The level 2 hierarchy of CAZY.


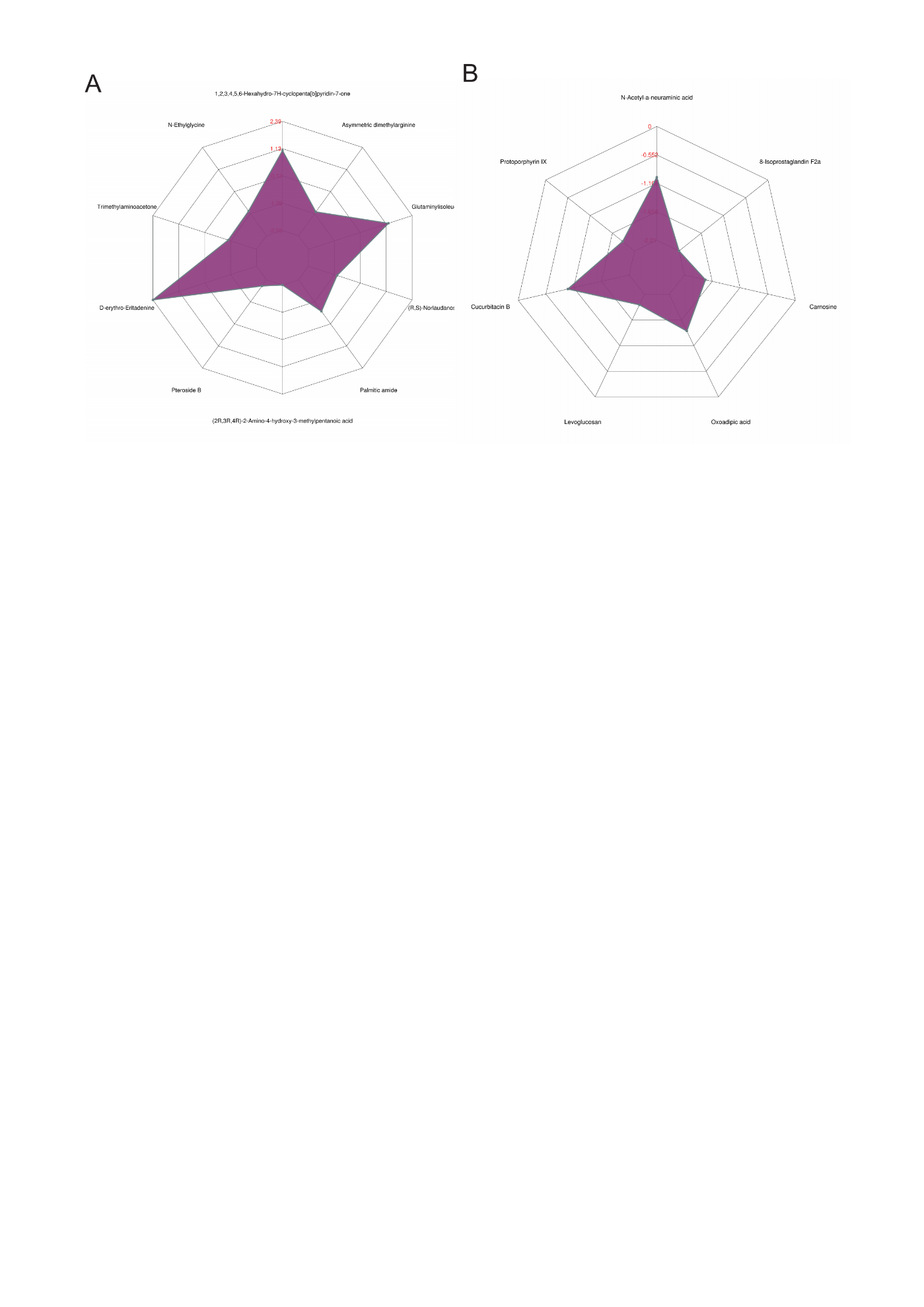


**Supplementary Figure 3.** The radarplots of DE metabolites. (A) The radarplot of DE metabolites under positive ion model. (B) The radarplot of DE metabolites under negative ion model.
